# The Role of Biomaterials in Upper Digestive Tract Transoral Reconstruction

**DOI:** 10.3390/ma14061436

**Published:** 2021-03-16

**Authors:** Raluca Grigore, Bogdan Popescu, Şerban Vifor Gabriel Berteşteanu, Cornelia Nichita, Irina Doinita Oașă, Gloria Simona Munteanu, Alexandru Nicolaescu, Paula Luiza Bejenaru, Catrinel Beatrice Simion-Antonie, Dragoș Ene, Răzvan Ene

**Affiliations:** 1 Otorhynolaryngology Department, Colțea Clinical Hospital, 917151 Bucharest, Romania; raluca.grigore@umfcd.ro (R.G.); serban.bertesteanu@umfcd.ro (Ş.V.G.B.); irinaoasa@gmail.com (I.D.O.); 2 Department 12-Otorhynolaryngology, Ophtalmology, Faculty of Medicine, “Carol Davila” University of Medicine and Pharmacy, 050474 Bucharest, Romania; gloriamunteanu@gmail.com (G.S.M.); alexandrunicolaescu@ymail.com (A.N.); drpaulabejenaru@gmail.com (P.L.B.); catrinel_antonie@yahoo.com (C.B.S.-A.); 3 3Nano-SAE Res Center, Faculty of Physics, University of Bucharest, 077125 Bucharest-Magurele, Romania; cornelia@3nanosae.org; 4 National Institute for Chemical-Pharmaceutical Research and Development, 031299 Bucharest, Romania; 5 Otorhynolaryngology Department, “Carol Davila” Emergency University Military Hospital, 010825 Bucharest, Romania; 6 General Surgery Department, Emergency Clinical Hospital, 917151 Bucharest, Romania; dragos.ene@umfcd.ro; 7 Department 10-General Surgery, Faculty of Medicine, “Carol Davila” University of Medicine and Pharmacy, 050474 Bucharest, Romania; 8 Orthopedics and Trauma Department, Emergency Clinical Hospital, 917151 Bucharest, Romania; razvan.ene@umfcd.ro; 9 Department 14-Orthopedics, Anaesthesia Intensive Care Unit, Faculty of Medicine, “Carol Davila” University of Medicine and Pharmacy, 050474 Bucharest, Romania

**Keywords:** malignant neoplasia, transoral reconstruction, polydimethyl siloxane, Ag nanoparticles, fatigue strenght, prosthesis

## Abstract

This study aims to establish whether the use of biomaterials, particularly polydimethylsiloxane (PDMS), for surgical reconstruction of the esophagus with templates, Montgomery salivary tube, after radical oncology surgery for malignant neoplasia is an optimal choice for patients’ safety and for optimal function preservation and organ rehabilitation. Structural analysis by Raman spectrometry and biomechanical properties with dynamic mechanical analysis are performed for fatigue strength and toughness, essential factors in durability of a prosthesis in the reconstruction practice of the esophagus. Nanocomposites with silicone elastomers and nanoparticles used in implantable devices and in reconstruction surgery present risks of infection and fatigue strength when required to perform a mechanical effort for long periods of time. This report takes into account the effect of silver (Ag) nanoparticles on the fatigue strength using polydimethylsiloxane (PDMS) matrix, representative for silicon elastomers used in implantable devices. PDMS with 5% (wt) Ag nanoparticles of 100–150 nm during mechanical fatigue testing at shear strength loses elasticity properties after 400 loading-unloading cycles and up to 15% shear strain. The fatigue strength, toughness, maximum shear strength, as well as clinical properties are key issues in designing Montgomery salivary tube and derivates with appropriate biomechanical behavior for each patient. Prosthesis design needs to indulge both clinical outcomes as well as design methods and research in the field of biomaterials.

## 1. Introduction

Silicone is used for head and neck implantable devices in the form of cochlear implants [1], nose implants [2], and prosthesis of the upper respiratory and digestive tract [3,4].

The main issue of implantable prosthesis is by far related to the physical and mechanical characteristics regarding strength, shear strain and longevity. For this issue to be addressed some improvements in the structure of the silicone polymers used have been made. Nanocomposites are the result of different molecules incorporated in the structure of the polymers [5,6] The development of such composite mixtures must increase the performance, utility, productivity and product uniformity [7,8], as well as to ensure optimal blend by polymer dispersion for improved properties [9].

Depending on the polymers used to increase the strength and shear strain different results can be achieved. In the head and neck region a silicone elastomer mixed with nano alumina ceramic fiber was used by Nouri Al-qenae for facial reconstruction. However, their results established that this mixture does not have significant outcome in terms of needed properties [10]. Nonetheless, Sara M. Zayed et al. and Dhuha A. Shakir and Faiza M. Abdul-Ameer conducted a series of mechanical testing regarding SiO_2_ nanoparticles, respectively TiO_2_ nanoparticles. Results showed that after incorporation all mechanical properties of the polymers improved [11,12].

More recent studies concerning silicone polymers revealed that 3D printed prosthesis is likely to suffice mechanical problems like viscosity when compared to the classical indirect molding technique, as described by Eric et al. [13].

Viscosity of the polymer tends to act inversely to strain hardening effect in terms of mechanical cycles, therefore less viscosity more cycles [14].

Malignant neoplasia of the upper digestive tract is subject to oncology therapy which implies surgery. In most cases, radical resection needs to be performed and the tissue defect needs to be replaced so that function can be restored. Several techniques have been imagined and used in this type of reconstruction. In the E.N.T. Clinic of Colțea Clinical Hospital, we developed a proprietary method which resides in the use of a prosthesis by which the upper digestive tract continuity is being re-established. 

Biomaterials used for reconstruction after oncology reconstruction must fulfill demands of biocompatibility ranging from simple biomechanical use to high bioaffinity with tissues and organs. In general, biocompatibility refers to implantable medical devices and organ replacements for reconstructive surgery. Multiple reviews and research focus on different types of biomaterials with specific relation to a targeted application. Hierarchically, the biomaterials must perform a main function (or more) with associated biocompatibility defined by local bioenvironment [15,16,17]. The esophagus may have the capacity to change its propulsive force in response to bolus size and neurohumoral agents [18]. Esophagus reconstruction surgery has a bioenvironment that is quite complex. The biomaterials used in reconstruction are in direct contact with tissues and local organs respective, food (bolus), beverages and saliva, whilst being in indirect contact with external environment. In such applications, they should fulfill several properties (Table 1).

**Figure 1:**
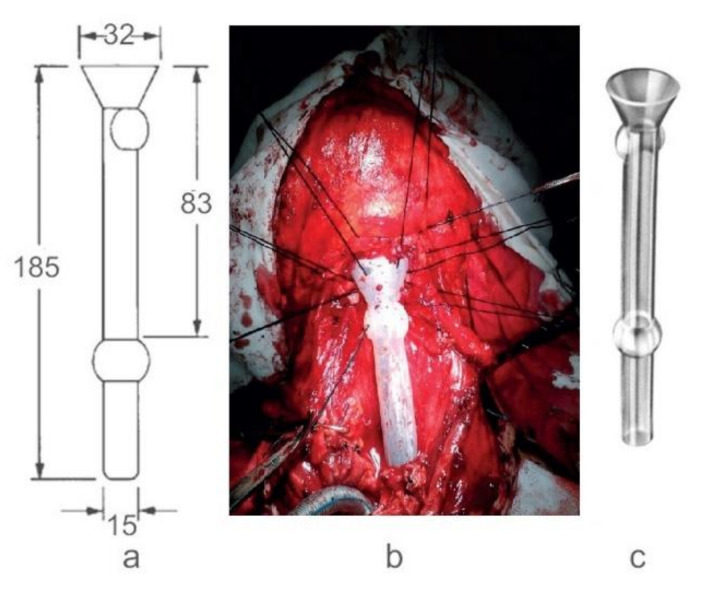
(**a**) Montgomery salivary tube, constructive principle (dimensions in mm) (**b**) A case study implemented by the Colțea Hospital ENT Team (iconography Dr. C.R Popescu [22]) (**c**) Montgomery tube made of silicon rubber.

Today, the most used templates for esophageal prosthesis are Montgomery salivary bypass tube [19,20,21], a viable alternative in reconstruction. In the tubular region, the prosthesis has two spherical zones (areas) which assure a better stability and optimal saliva leaking along of tube, independent of head and neck position (Figure 1).

In the range of silicon rubbers, polydimethylsiloxanes (PDMS) have several advantages in designing esophageal prosthesis: it can be molded in different shapes in vacuum or by centrifugation for degassing; the ratio silicon resins/hardener (curing agent) can be adjusted to elaborate a cross-linked rubber with convenient biomechanical properties: molecular weight, viscosity, elasticity related to the shear forces; the PDMS surfaces can be treated (such as plasma treatment) to obtain superhydrophobic/hydrophilic properties or to insert various antioxidants and bacteriostatic agents (addition of Ag). The biofilm represents a sessile microbial community comprising microbial cells with altered phenotype, characterized by a reduced growth rate and altered gene expression [23]. Biofilms are generally included in a protective polysaccharide matrix secreted by biofilm cells and are found attached to a surface [24,25,26,27,28,29].

In addition, by designing appropriate composites with different nanomaterials PDMS can be radio-opaque or radio-transparent for locally induced radiotherapy. Moreover, it can be designed for appropriate insertion of biopolymers containing various drugs with controlled release for local therapy.

One key issue with PDMS is biomechanical stability during working conditions, such as fatigue strength, maximum strain deformation, storage and dissipation modulus. These characteristics should be accommodated with the biomechanical properties of each patients’ tissue particularities where one prosthesis replaces one organ.

This study reviews the mechanical behavior during cyclic loading-unloading by simulating the bolus dynamics (fatigue strength, toughness, shear strain) for the in vitro part of the study and reviews the parameters of clinical outcome in terms of biocompatibility, functionality and antibacterial and antifungal properties of the PDMS template with Ag addition. Ag nanoparticle PDMS templates used for surgical reconstruction of the hypopharynx and cervical esophagus help surgeons and improve the quality of life for patients, since this type of surgery is in most cases a challenge for the oncology head and neck surgeon [22,30,31,32].

This study aims to establish whether the use of biomaterials, particularly polydimethylsiloxane (PDMS) with Ag nanoparticles, for surgical reconstruction of the esophagus with templates, after radical oncology surgery for malignant neoplasia is an optimal choice for patients’ safety and for optimal function preservation and organ rehabilitation. Esophagus prosthesis used for upper digestive tract reconstruction are prone to fungi colonization, in most cases with *Candida albicans*. One of our aims during this study was to establish whether silicone elastomers mixed with silver nanoparticles present antifungal properties.

## 2. Materials and Methods

Our design for the study encompassed: preparation of PDMS samples for testing, reference and silicone-silver nanoparticle polymers, Raman spectrometry to determine the chemical structure of the polymer, mechanical analysis of the samples and comparison analysis, and in vitro testing of prosthesis for biocompatibility and clinical surgery.

### 2.1. Sample Preparation

We designed a study to compare the use of biomaterials in hypopharynx and cervical esophagus transoral reconstruction, with or without mandible reconstruction. For this we used a reference sample, transparent Montgomery esophageal tube (Boston Medical Product, Inc, Shrewsbury, MA, USA) and PDMS recipes, described as following. There is a large class of siloxane base oligomers and associated curing agents. Similar to silicon rubber used in Montgomery salivary tube (Boston Medical Products, Inc.) there are series of other silicon rubber used in designing components for microfluidics and soft lithography such as Sylgard 182–186 (Dow Corning, Midland, MI, USA).

After curing, the elastomers are translucent and have various mechanical properties following smart design with appropriate ratio silicon oligomers/curing agent. Usually, the recommended ratio is 10:1 and the curing temperature up to 150 °C. Slight variations in silicon oligomers/curing agent ratio means that the mechanical properties are well reproduced as Montgomery salivary tube. Samples were tested in the shear stress conditions.

The S1 sample was taken from Montgomery Salivary tube (as specified in Figure 1). S1a- samples were prepared from Sylgard 184 to match the resistance properties of the S1 sample and similar mechanical properties. S2-samples were prepared with Sylgard 184 with a specific ratio oligomer/curing agent (10:1) to obtain similar mechanical properties as S1. S2 was prepared by mixing the Sylgard 184 oligomer with silver (Ag) nanopowder (transmission electron microscopy-TEM diameter ~100–150 nm, average hydrodynamic diameter ~230 nm, measured by dynamic light scattering-DLS) in 5% (wt/wt) and then adding curing agent and treated at 130 °C for 30 min. Silver nanoparticles were prepared using specific methods [33]. All samples were shaped so they could form test specimens of 10 mm diameter and 1mm thickness.

### 2.2. Raman Spectrometry

Raman spectrometry used for data spectral data analysis included Jasco, NRS-3100 (Easton, PN, USA) with dual laser beams, 532 and 785 nm, resolution 4 cm^−1^, with specific configuration and backscattering.

Raman analysis is performed to acquire data from a chemical point of view. We performed the spectral analysis to quantify the number of repeatable units in PDMS—rubber, to establish PDMS network with cross-linkage on –Si–O–Si– and to verify cross-linked PDMS-network via vinyl groups.

### 2.3. Mechanical Analysis

**Figure 2:**
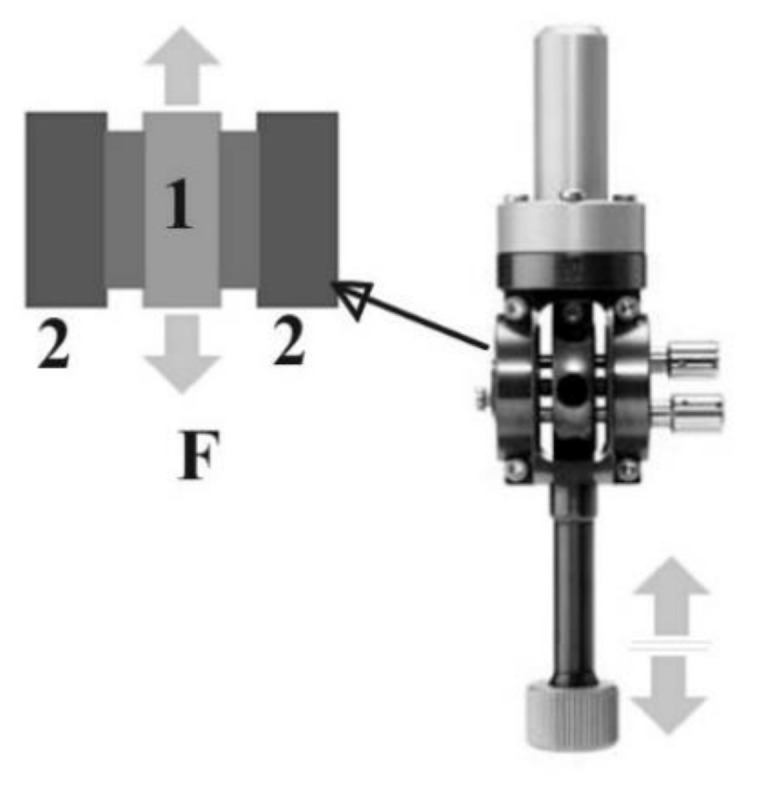
Configuration for shear operating mode in DMA/SDTA 861, STAR SYSTEM, produced by Mettler Toledo. 1-mobile plate, 2-fixed back support. F-shear force.

Mechanical analysis was performed using Dynamic mechanical analyzer/Simultaneous thermal analysis DMA/SDTA861, STAR SYSTEM, produced by Mettler Toledo (Greifensee, Switzerland). The operating mode implied Shear function (shear force vs shear strain). This mode is ideal for elastomers, thermo-plastic materials and thermosets. In the shear mode, two identical samples were clamped symmetrically between two fixed outer parts (2) and a central moving part, (1) (Figure 2). The shear clamp guarantees a homogeneous temperature distribution. A thermocouple mounted directly in the clamp measures the samples’ temperature so precisely that simultaneous heat flow effects could also be determined (SDTA). The shear force F ranged from 1 mN to 40 N, frequency ranged from 1 mHz to 1000 Hz. The sample diameter had a maximum ≤14 mm and the thickness had a maximum ≤6.5 mm. The experimental set-up included a sample of 10 mm in diameter, thickness of 1 mm, with 1Hz frequency, at room temperature. Maximum shear strain for the samples investigated were 100 microns, respective 150 microns. That corresponded to the increase in diameter of the Montgomery tube at around of 10%, respective 15%.

### 2.4. Clinical Surgery

After in vitro testing of the new biocompatible prosthesis, we performed clinical testing. Clinical testing included accurate selection of patients according to clinical inclusion criteria (age above 18 years of age, informed consent of the patient, malignant neoplasia of the upper esophagus, esophagectomy prior to esophagus reconstruction, no radiotherapy as adjuvant therapy). Exclusion criteria were the absence of one or more of the above-mentioned inclusion criteria or the preference of the patient to leave the clinical study. No such aspects were encountered. The publication of surgical therapy and clinic trial results were approved by The Ethical Committee of Coltea Clinical Hospital according to decision 20911/05.11.2020. Patients have been distributed into two groups, one of 21 patients treated by using the original Montgomery tube and the second one including 18 patients treated by using PDMS Ag-nanoparticle implant.

Colţea ENT Clinic used an original implantation technique of the Montgomery prosthesis that met the criteria for optimal reconstruction: refueling facility, oral method, reduced complications and mortality, period of hospitalization with lower costs [22]. In selected cases, where mandible reconstruction was needed, a multidisciplinary team of general surgeon and orthopedic surgeon was required [34,35].

The surgical intervention was performed in all cases under general anesthesia and oro-tracheal intubation. The removal of the larynx, part of the hypopharynx, and the cervical esophagus was the main surgical act, and it was performed since all patients included in the clinical study were diagnosed with malignant neoplasia of the hypopharynx and cervical esophagus. The resection of the larynx is included in the surgery protocol since both the hypopharynx and the cervical esophagus cannot be removed without larynx removal for this type of cancer patients. Reconstruction was made by using the original Montgomery tube for group 1 and PDMS Ag-nanoparticle implant for group 2. The insertion of the prosthesis was performed transoral for better fitting at the base of the tongue. Surgery was completed by reconstructing the muscle, subcutaneous and skin tissues. A naso-gastric feeding tube was placed through the prosthesis to ensure feeding until healing was completed.

Follow-up included the immediate postoperative period of 14 days at the end of which sutures were removed and oral feeding was restarted. Late follow-up included scheduled visits of patients for endoscopic examination every other 2 months. Cervical computer tomograph evaluation was performed at an interval of 6 months for a period of 2 years after surgery.

## 3. Results

### 3.1. Raman Spectroscopy

**Figure 3:**
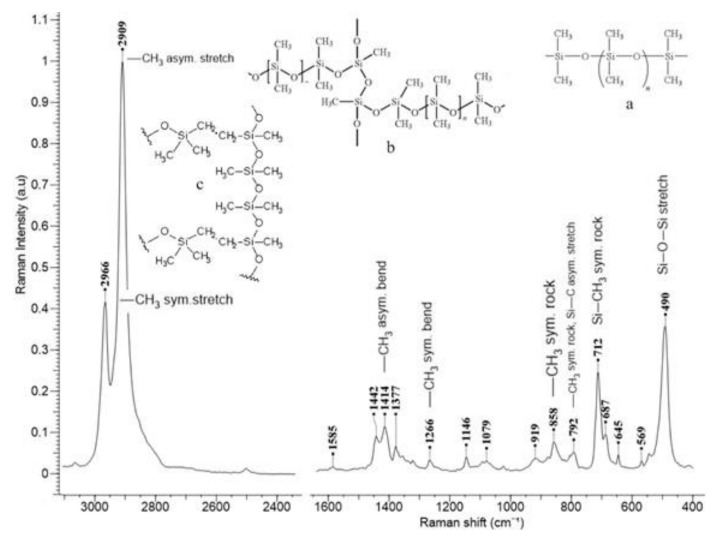
Raman spectrum recorded for S1, (**a**) the repeatable unit in PDMS—rubber, (**b**) PDMS network with cross-linkage on –Si–O–Si–, (**c**) PDMS-network cross-linked via vinyl groups.

Raman spectrometry used for data spectral data analysis included Jasco, NRS-3100 with dual laser beams, 532 and 785 nm, resolution 4 cm^−1^, with specific configuration and backscattering, averaging over 1000 spectra giving the confidence interval. Error is under 0.1%. Raman spectroscopy gives a strong information related to the structural modifications and it is not appropriate for statistical analysis. Statistical analysis should be performed only in case there are strong variations in structure. Raman spectra for samples S1 and S1a show specific features for PDMS-rubber. For exemplification, the spectrum is recorded for S1 (Figure 3).

The spectrum shows several features specific for simple repeatable units (Figure 3a). We identified several bands assigned to –CH_3_ groups respective to the backbone –Si–O–Si–. They are well defined and have high intensity. The other bands with weak intensity are associated with a particular type of cross-linkage developed in PDMS network: 1585 cm^−1^ (C=C, in phase-stretching), 1442 cm^−1^ (δ(CH_2_) scissoring), 1377 cm^−1^ (–CH_3_, methyl rocking), 1146 cm^−1^ (C–C stretching), 1079 cm^−1^ (in-plan rocking for C=CH group). In conclusion, the polymer develops a network with cross-linkage on –Si–O– (Figure 3b) with low levels of cross-linking on –CH_2_–CH_2_– vinyl group (Figure 3c).

### 3.2. Shear Stress/Shear Strain

**Figure 4:**
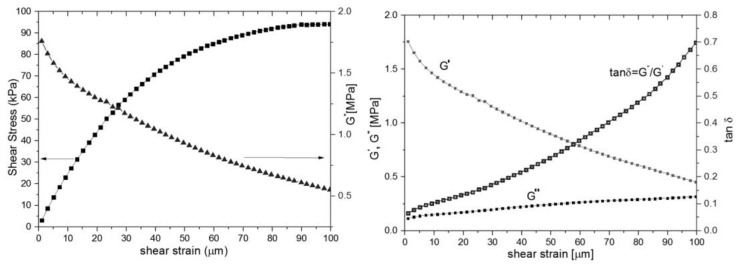
Mechanical properties measured by DMA, sample S1, maximum shear strain 100 microns: (**left**) shear stress vs shear strain and G^*^-shear modulus; (**right**) storage (G′), dissipative modulus (G′′) and loss tangent tanδ.

Mechanical analysis was performed using DMA/SDTA861, STAR SYSTEM, produced by Mettler Toledo. The mechanical properties measured in shear mode are shown in Figure 4 for samples S1 and S1a. For the same reason we show results for S1.

The shear stress/shear strain curve has a quasilinear behavior up to 30 microns per millimeter reaching a plateau at shear strain ~100 microns (Figure 4 left). This is consistent with rubber-elastic materials and their capacity to reach a “rubbery plateau” with quasi-reversible return in an initial state [36]. The shear modulus (Figure 4, left) is dependent of shear strain, which is typical for a viscoelastic rubber material. The shear modulus (G*) varies from 1.8 to 0.5 MPa. The loss modulus (G”) increases slightly with the shear strain, therefore the dissipative energy during loading, mainly bolus transition, reaches at ~1–2 mJ. Even though the storage modulus (G’) involved in elastic energy recovery decreases with the strain deformation, PDMS still has a good capacity to regain the initial state after deformation (Figure 4, right). The loss tangent continuously increases at values less than unit without any phase changes. That is consistent with the behavior of quasi-elastic materials with high flexibility.

### 3.3. Fatigue Strength

The statistical method we used is one-way analysis of variance (ANOVA) with mean, standard deviation, standard error, F-statistic value and *p*-value determinations. Each group of the S1 (Montgomery tube), S1a (Sylgard 184) and S2 (Sylgard 184 with Ag nano-powder) polymers consisted of 20 samples for testing and statistical analysis. The statistical analysis was aimed on comparing the properties of S1 and S1a samples and S1a and S2 samples. S1a sample was manufactured from Sylgard 184 to match the characteristics of the S1 sample since we used Montgomery tube as reference. S1a/S2 comparison was performed after establishing the similarities of S1a to S1 (Table 2 and Table 3).

**Figure 5:**
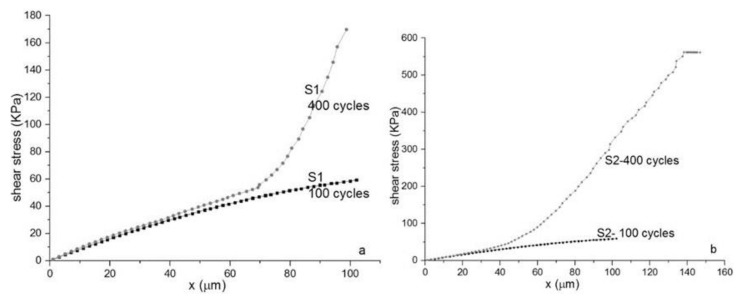
Mechanical tests: Fatigue strength in shear mode. (**a**) Sample S1, (**b**) Sample S2. Samples are loaded up to 100 µm and unloaded at shear strain rate 0.1 µm/s. Maximum shear strain 100 µm. Toughness increases with the number of cycles tested to fatigue strength. S2-Ag nanoparticles decreases toughness.

Samples S1, S1a and S2 are cyclically loaded and unloaded up to 150 microns, their maximum shear strain. After each 100, respective 400 cycles each sample is mechanical tested at their maximum shear strain of 100 microns. After 100 cycles samples S1 keep a rubber-elastic shear strength behavior with a rubbery plateau decreased from 90 KPa to 60 KPa (Figure 5). After 400 cycles, the sample S1 keeps elastic properties in the range of shear-strain up to 70 microns. At higher shear deformation, S1 has quite a different behavior with increased toughness. It can be hypothesized that PDMS network, during cyclic loading-unloading, reinforces by increasing self-cross-linkage between dangling bonds. Similar behavior is observed for sample S2. The elastic state reduces up to 40 microns shear strain and after this value the reinforcing increases more prominent than S1.

As shown in Table 2, there is no statistical difference between S1a and S1 samples which is consistent with similar properties between the two. We were looking for a Sylgard 184 sample with the closest properties to Montgomery tube. Therefore, the share-strain curve in linear viscoelastic region for both S1 and S1a samples is similar.

As shown in Figure 5 and Table 3 there is a mechanical difference between S1/S1a samples and S2 samples. However, the logarithmic regression of the share-strain curve in linear viscoelastic region for the two different type of samples is similar. Statistical analysis showed that there is no statistical differences between the two groups (*p*-value = 0.1104).

### 3.4. Clinical Testing

We compared the PDMS Ag-nanoparticle implant to the original Montgomery tube used for esophagus reconstruction. While 21 cases were treated by using the original Montgomery tube some 18 cases underwent esophagus reconstruction using PDMS Ag-nanoparticle implant. In terms of biocompatibility and functionality the results were similar with a *p*-value of 0.0012, respectively 0.0004, were as for antibacterial and antifungal purposes the PDMS Ag-nanoparticle implant performed better since only 2 *Candida albicans* included biofilms have been detected at 12 months after surgery. In comparison, the original Montgomery tube developed on the inner surface *Candida albicans*, *Staphylococcus aureus* and *Actinomyces* spp. in 17 out of 21 cases (*p* = 0.42) (Table 4).

**Figure 6:**
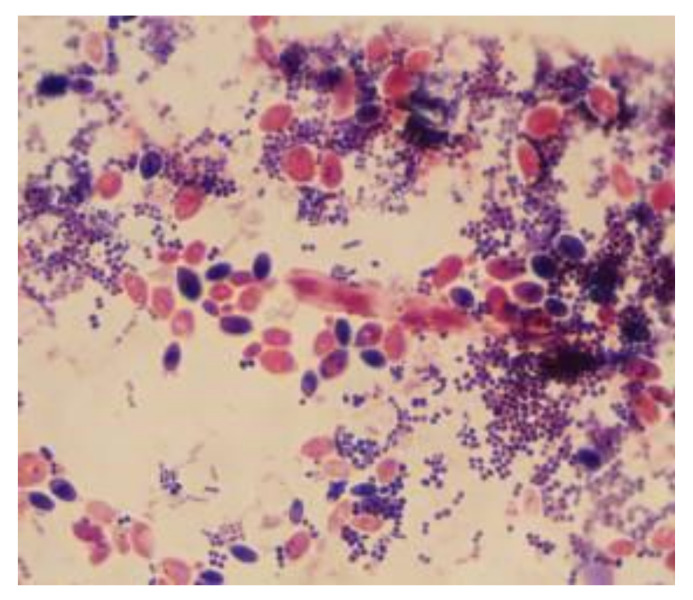
Microscopic images of Gram-stained smears performed directly from the biofilm developed on the surface of the voice prosthesis (Gram stain, ×1000).

**Figure 7:**
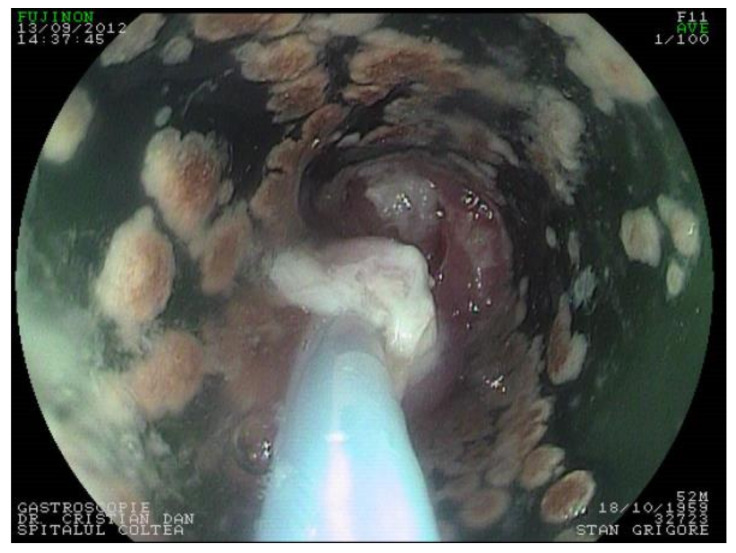
Biofilm, inlcuding *Candida* spp., *Staphilococcus aureus*, forming on the inner surface of the prosthesis.

**Figure 8:**
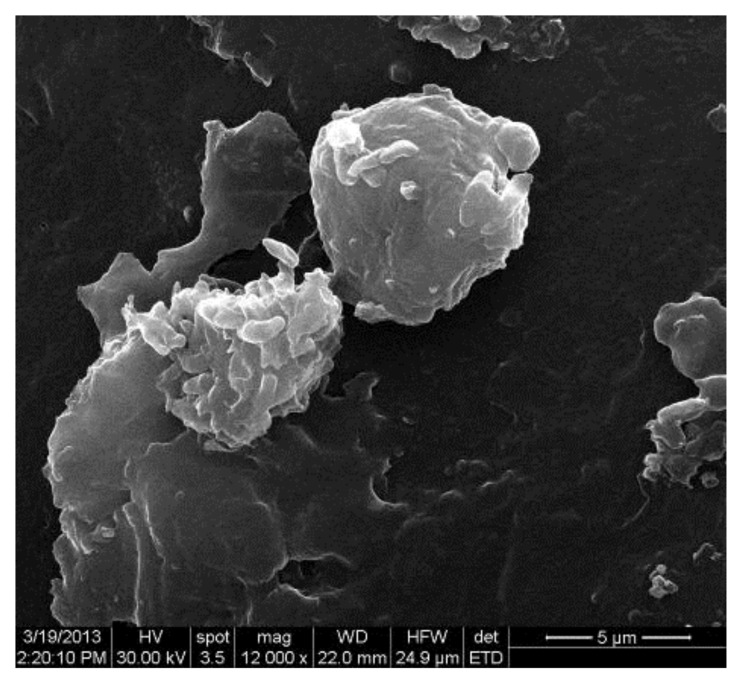
Electron-microscopy images of microbial biofilm developed on the surface of the voice prosthesis.

The microscopic examination of Gram-stained smears performed directly from the biofilm developed on the surface of the voice prosthesis revealed the presence of a frequent association between different morphological types, indicating the specific nature of microbial biofilm (Figure 6 and Figure 7). The examination of the colonized esophageal prosthesis specimens by scanning electron microscopy (SEM) revealed the presence of a mature biofilm, with a consistent matrix and a complex three-dimensional structure (Figure 8). The microscopic examination of different regions of the prosthetic device has shown that biofilm deposits are accumulating along the entire surface of the device. The well-developed biofilm could therefore be responsible for the prosthesis dysfunction and limited resistance over time.

The follow-up period for the clinical part of the trial was 12 months for PDMS Ag nanoparticles prosthesis. Oncology status was followed-up for 24 months and was a secondary aspect of the clinical trial and with no relation to the aim of the study. However, the statistical analysis we performed in the ENT department of Colțea Clinical Hospital, related to the different forms of relapses of hypopharynx and cervical esophagus cancer concluded that only 34% of patients did not have a relapse of their cancer.

## 4. Discussion

The study describes the first prosthesis design using a combination of silicone elastomer and silver nanoparticles used for the reconstruction of the upper digestive tract. This project was based on the previously used Montgomery prosthesis which is a single silicone polymer device. Our main goal was to create an adequate polymer which can support the functions of the previous Montgomery prosthesis improved in terms of fungus colonization. Clinical outcome is dependent on the structural design of the prosthesis and on the capability of the biomaterial.

Along with the technique used to surgically implant the prosthesis a concurrent well-known problem appeared. Biofilm formation was observed in sample prosthesis. Infections resulting from microbial adhesion to medical surfaces have been observed on all prosthetic devices, regardless of the biomaterial nature, with severe economic and medical consequences. Biofilm-associated infections raise some clinical challenges, including disease, chronic inflammation, and rapidly acquired antibiotic resistance [37]. We compared clinical outcome in terms of biotolerance, functionality, and inner surface biofilm formation of PDMS Ag-nanoparticles implants with the original Montgomery tube.

Silicone based polymers have been used for reconstruction in the head and neck region but failed to address all the issues. Silver nanoparticles have proven their role in diminishing germ and fungus colonization of biomaterials. The blend that we used to develop the improved Montgomery prosthesis is similar to other blends that have already been used in biocompatible devices. Therefore, the method is already having its applications.

Raman spectrometry and mechanical testing provided us with basic and advanced data regarding the functional parameters of the prosthesis and these are consistent to other studies involving silicone/silver nanoparticles biomaterials [36]. By using this blending technique and receipts the polymer develops a network with cross-linkage on –Si–O– with low levels of cross-linking on –CH_2_–CH_2_– vinyl group which confers high levels of elasticity and, thus the possibility of high elastic energy accumulation.

Given the fact that the shear stress/shear strain curve has a quasilinear behavior up to 30 microns per millimeter reaching a plateau at shear strain ~100 microns we were able to say that this polymer is consistent with rubber-elastic materials. This behavior enables the polymer to return, after a deformation, to a quasi-reversible return to the initial state. All the data recovered from the shear stress/shear strain curve leads to the fact that this polymer has high flexibility which is mandatory for this type of prosthesis use.

Both samples, S1 and S2, interpreted in terms of toughness, show several features useful in biomechanical properties. In physical sense, toughness is really a measure of the energy absorbed before it breaks and estimated from the area underneath the stress-strain curve. Both samples show a transition from low toughness (high rubbery state with low energy absorbed) to high toughness where adsorbed energy is high and leads to breaking, losing the essential properties during the effort done for bolus transition.

In terms of quality of life, the reconstruction with synthetic prosthesis for most patients is well tolerated, considered satisfactory regarding silicone implant. In comparison to the original Montgomery tube, the PDMS with silver-nanoparticles has increased utility in terms of antibacterial and antifungal biofilm formation. The clinical results as shown on cohorts of patients after prosthesis implant and treated by a multidisciplinary approach, respective by monotherapy show a relatively good biocompatibility of the silicon rubber blended with silver nanoparticles [38,39,40].

To ensure that cytotoxicity due to silver impregnation is not posing any risks for our patients we determined the total silver levels in vitro which were below admitted levels (<1.1 µg L^−1^). These data are consistent with results from a study made by Zhala Meran et al. who stated that prosthetic materials coated with silver nanoparticles are biocompatible with fibroblast cells [41]. According to SCHEER guidelines Montgomery prosthesis silicone elastomer mixed with silver nanoparticles meet the criteria for tolerable exposure (EN ISO 10993-17:2002) and for analytical contact conditions (EN ISO 10993-12) [42].

The limitation of the reconstructive method is derived from the inconsistency between mechanical properties of the Montgomery tube (given mechanical properties) and biomechanics of the esophagus for each patient. There is a wide variety of individual characteristics concerning anatomy landmarks and function preservation. The rehabilitation using this technique cannot overcome the prior functional status of each patient, hence the variation in function preservation. However, clinical outcome in terms of biocomplatibility, functionality, and biofilm formation prevention over repeated cycles of use for the Montgomery tube coated with silver nanoparticles showed that the rehabilitation method and the characteristics of the improved prosthesis are optimal for upper digestive tract reconstruction.

## 5. Conclusions

Silicon rubbers used in prosthesis for a specific surgery application (esophagus reconstruction) are the most appropriate solution in terms of morbidity, biocompatibility, functionality and bacterial and fungal biofilm formation.

The mechanical properties can be tailored by optimal recipes silicon oligomers/curing agent and curing temperatures, as shown previously. The fatigue strength, toughness, maximum shear strength are the key issues in designing Montgomery salivary silicone tube blended with silver nanoparticles with appropriate biomechanical behavior for each patient. Insertion of bacteriostatic agents, such as silver nanoparticles, decreases the fatigue strength, increases flexibility and offers optimal local protection solution against fungi development.

Furthermore, starting from the premises that including nanoparticles of different agents is a real possibility, we can state that this finding is useful in imagining a new concept to use bacteriostatic agents or other drugs for local therapy.

Prosthesis design needs to indulge both clinical outcome as well as design methods and research in the field of biomaterials. Transoral insertion of a prosthesis for esophagus reconstruction after cancer surgery has improved overall survival rates and the quality of life for these patients.

Further data and analysis will show eventual pitfalls of the technique.

## 6. Patents

Montgomery esophageal prosthesis are coded 878.3610 by Code of Federal regulations (21CFR 878). It is made of biocompatible silicon (recommended by Blue Book G95-1: ISO-10993 Biological Evaluation of Medical Devices Part1) tested for cytotoxicity, sensibility, implantation and sub-chronic and chronic toxicity. The reconstructive method is subject to Romanian patent for medical devices no. 130466 from 19.08.2014.

## Figures and Tables

**Table 1:** Properties of artificial esophagus used for reconstruction surgery.

Properties of Artificial Esophagus	Application Requirements Fulfillment
Biomechanical	Related to the stress-strain mechanical response Optimized storage modulus for organ biomechanics Optimal dynamics for propulsive forces related to bolus size
Tribological (inner surface)	Self-cleaning Hydrophobic compatibility with saliva and bolus dynamics
Thermo-physical	Low swelling related to biological fluids and water-intake Chemically inert Non-biodegradable
Bacteriostatic and antifungal	Inner lining bacteriostatic and antifungal activity
Connectivity to other organs	Biocompatibility with the surrounding tissues
Electroactivity	Responsiveness to neurohumoral agents

**Table 2:** Comparison of compression modulus of S1 and S1a samples as obtained by the slope of the share-strain curve in linear viscoelastic region.

Data Summary					
Groups		N	Mean	Std. Dev	Std. Error
Group 1		20	49.8795	1.1082	0.2478
Group 2		20	49.034	0.7865	0.1759
**ANOVA Summary**					
**Source**	**Degrees of Freedom DF**	**Sum of Squares SS**	**Mean Square MS**	**F-Stat**	***p*-Value**
Between Groups	1	7.1487	7.1487	7.7422	0.0084
Within Groups	38	35.0871	0.9233		
Total	39	42.2358			

**Table 3:** Comparison of compression modulus of S1a and S2 samples as obtained by the slope of the share-strain curve in linear viscoelastic region.

Data Summary					
Groups		N	Mean	Std. Dev	Std. Error
Group 1		20	51.7685	1.3128	0.2935
Group 2		20	51.036	1.5143	0.3386
**ANOVA Summary**					
**Source**	**Degrees of freedom DF**	**Sum of Squares SS**	**Mean Square MS**	**F-Stat**	***p*-Value**
Between Groups	1	5.3656	5.3656	2.6717	0.1104
Within Groups	38	76.3144	2.0083		
Total	39	81.68			

**Table 4:** Comparison of clinical parameters of original Montgomery tube and PDMS with silver-nanoparticles implant.

Samples	Montgomery Tube Cases	PDMS Ag-Nanoparticle Implant Cases	*p*-Value
Biocompatibility	20/21	18/18	0.048
Functionality	20/21	18/18	0.048
Antibacterial/Antifungal action	4/21	16/18	0.42

## Data Availability

Data available on request due to privacy and ethical restrictions concerning patients’ personal data. The data presented in this study are available on request from the corresponding author since main data is contained within the article.
